# 

**Published:** 1854-01

**Authors:** 


### CONTENTS.

ORIGINAL COMMUNICATIONS:— PAGE

ART I.—On the Uses of Turpentine. . . . . . . . . 1

ART II.—Gunshot Wound. By S. O. Long, M. D. . . . . . . 2

SELECTIONS:—

Notes of Travel. . . . . . . . . . . . . . 9

Are the Subjects of Convulsion and of Anesthesia conscious? . . . 13

Case of Precocious Development of the Sexual System in a Female Child. 15

Silk instead of Sponge for Laryngeal Peobangs. . . . . . . . 16

Alum as an Emetic. . . . . . . . . . . . 16

Traumatic Emphysema. . . . . . . . . . . 17

Elimination of Lead by Iodide of Potassium. . . . . . . 20

External Stimulus in Cholera. . . . . . . . . . . 21

Aphonia of Four Years' Standing, cured by Electro-Magnetism. . . 22

Case of Poisoning by Opium. . . . . . . . . . . 22

Extract from a Report of the Standing Committee on Surgery, read before the Kentucky State Medical Society Oct. 1853. . . . . 23

EDITORIAL:—

A Treatise on the Venereal Disease. . . . . . . . . . 25

Experimental Researches applied to Physiology and Pathology. . 36

An address delivered before the Pennsylvania Society of Dental Surgery. 41

Medical Jurisprudence. . . . . . . . . . . 43

Knox County Medical Society. . . . . . . . . . 45

The American Medical Association. . . . . . . . . . 46

A Practical Treatise on Diseases of Children. . . . . . . 47

General Therapeutics and Meteria Medica. . . . . . . . 47

The Practice of Surgery. . . . . . . . . . . 47

To the Editors of the North-Western Medical and Surgical Journal. 48

